# Low neonatal blood glucose levels in cesarean-delivered term newborns at Khartoum Hospital, Sudan

**DOI:** 10.1186/1746-1596-9-112

**Published:** 2014-06-09

**Authors:** Shahad M Hussein, Yasir Salih, Duria A Rayis, Jalal A Bilal, Ishag Adam

**Affiliations:** 1 Department of Obstetrics and Gynecology, University of Khartoum, Khartoum, Sudan; 2 College of Medicine, Qassim University, Buraydah, Kingdom, Saudi Arabia; 3 Faculty of Medicine, University of Khartoum, Khartoum, Sudan

**Keywords:** Cord, Glucose, Cesarean delivery, Newborn, Sudan

## Abstract

**Abstract:**

**Background**

Glucose is the main source of energy for organ function in neonates. There are few published recent data on neonatal glucose levels during cesarean delivery.

**Methods**

A case (cesarean delivery) -control (vaginal delivery) study was conducted at Khartoum Hospital Sudan to compare blood glucose levels of term newborns born after elective cesarean delivery with those born vaginally.

**Results**

Cord blood glucose levels at delivery were significantly lower in women who had a cesarean delivery compared with those who delivered vaginally (99.8 ± 20.6 vs. 106.8 ± 11.1 mg/dl, P = 0.026), but there was no significant difference (97.8 ± 16.7 vs. 102.1 ± 9.6, P = 0.110) in newborn glucose levels at 2 hours after delivery between the groups. In linear regression, cesarean delivery (-6.475 mg/dl, P = 0.013) and maternal blood glucose levels at the time of delivery (+0.619 mg, P < 0.001) were significantly associated with mean cord glucose levels.

**Conclusion**

This study shows that cord blood glucose levels are significantly lower in cesarean-delivered neonates than vaginally-delivered neonates. In addition, cord blood glucose levels are significantly associated with cesarean delivery and maternal blood glucose levels at delivery.

**Virtual Slides:**

The virtual slide(s) for this article can be found here: http://www.diagnosticpathology.diagnomx.eu/vs/2011479878124993

## Letter to the Editor

There has been a recent dramatic increase in the rate of cesarean delivery [1,2]. There are many fetal and perinatal complications of cesarean delivery e.g. obesity, allergies, metabolic disturbance, and lower blood glucose levels in the offspring [3-6].

There are few published recent data on neonatal glucose levels during cesarean delivery [6-8]. Glucose is the main source of energy for organ function in neonates. In particular, glucose is an exclusive source of energy for the function of the central nervous system in neonates [9]. The definition and management of neonatal hypoglycemia are controversial and there is no constant cut-off point for low levels of neonatal glucose [10].

A case–control study was conducted at Khartoum Hospital, Sudan during April to June 2012 to investigate glucose levels in neonates born to women who delivered by elective cesarean. Cases were women delivered by elective cesarean (before labor) and controls were consecutive vaginal deliveries. In both arms of the study, women were at term (37–41 completed weeks of gestation), newborns were ≥ 2500 g at birth, there was no history of fetal problems, and Apgar scores were 8 or higher at 1 and 5 minutes.

Newborns of mothers with any medical disorder, ante/intra-partum complications, newborns with signs suggestive of perinatal stress, and instrumental delivery, and those who required intensive resuscitation and care were excluded from both cases and controls.

Mothers who underwent cesarean delivery were fasting for at least 6 h before cesarean delivery. All vaginally-delivered infants were placed at the breast immediately after delivery. Every newborn had a venous cord blood sample obtained from the umbilical cord and another sample was taken 2 h later from a peripheral vein.

This study received ethical clearance from the Research Board of the Faculty of Medicine, University of Khartoum, Sudan.

After signing an informed consent, demographic data were collected using a pretested questionnaire. Then maternal blood glucose levels at delivery and at 2 hours after delivery, cord glucose levels at delivery and 2 hours after delivery were measured immediately after collection for whole blood glucose levels using the bedside device Accu-Chek™ Multiclix (Roche Diagnostics, Mannheim Germany). Start of feeding time, sex of the newborn, neonatal weight, Apgar score, duration of labor, time of mother’s fasting, time of starting feeding after delivery, and fluids during labor, were recorded and analyzed.

Data were analyzed using SPSS for windows. Means and proportions of the basic socio-demographic, clinical data and blood glucose levels were compared between women who delivered vaginally and those who had a cesarean delivery using the Student’s t-test and *X*^2^ test, respectively. Linear regression was performed where blood glucose levels (cord, neonatal) were the dependent variable, and other variables (maternal and perinatal) were the independent variables. P < 0.05 was considered significant.

The two groups (55 women in each arm) were well matched in their basic characteristics (Table 1). The time of fasting was significantly longer in women who delivered by cesarean than in those who delivered vaginally (8.4 ± 1.1) vs. 3.4 ± 1.4 hours, P < 0.001, Table 1).

**Table 1:** Comparison of characteristics of women who delivered vaginally and by cesarean in Khartoum, Sudan

**Variables**	**Vaginal delivery**	**Cesarean delivery**	**P**
	**(n = 55)**	**(n = 55)**	
*The mean (SD) of*			
Age, years	28.2 (4.5)	29.0 (4.0)	0.323
parity	1.6 (1.4)	1.7 (1.3)	0.892
Gestational age, weeks	38.5 (1.0)	38.8 (1.0)	0.122
Body mass index, kg/cm^2^	28.1 (1.7)	28.3 (2.1)	0.725
Fasting time, hours	3.4 (1.4)	8.4 (1.1)	< 0.001
*Number (percentage) of*			
Educational level ≤ secondary	28 (51.0)	24 (43.6)	0.276
Rural residence	11 (20.0)	11 (20.0)	0.549
Lack of antenatal care	1 (1.8)	1 (1.8)	0.752
Normal saline received	53 (96.4)	46 (83.6)	0.052
5% dextrose received	2 (3.6)	9 (16.4)	

**Figure 1:**
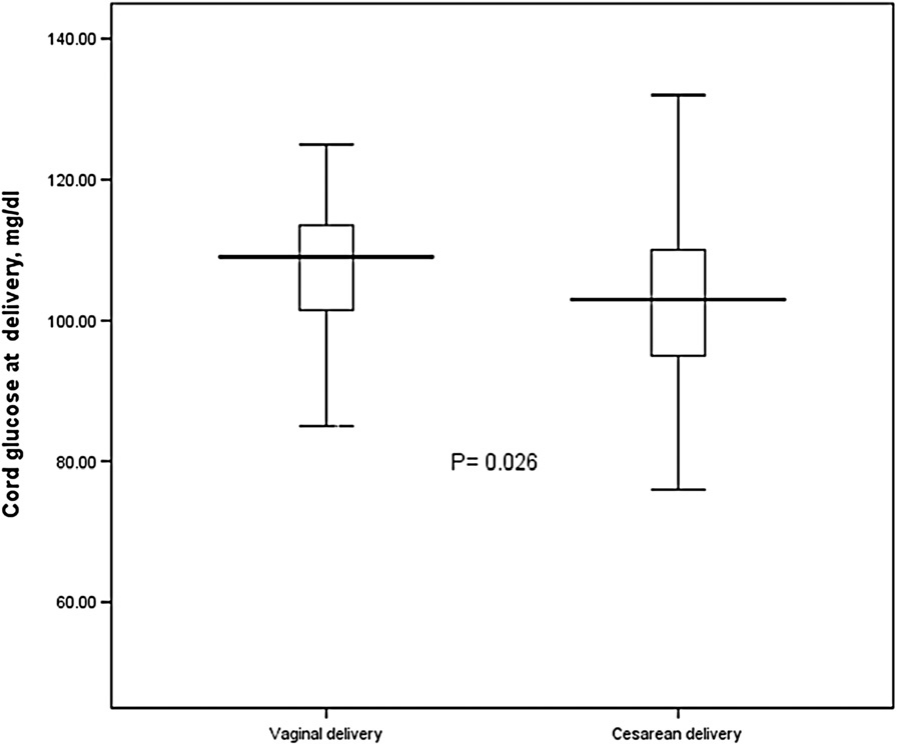
Cord blood glucose levels in vaginal and cesarean deliveries.

**Figure 2:**
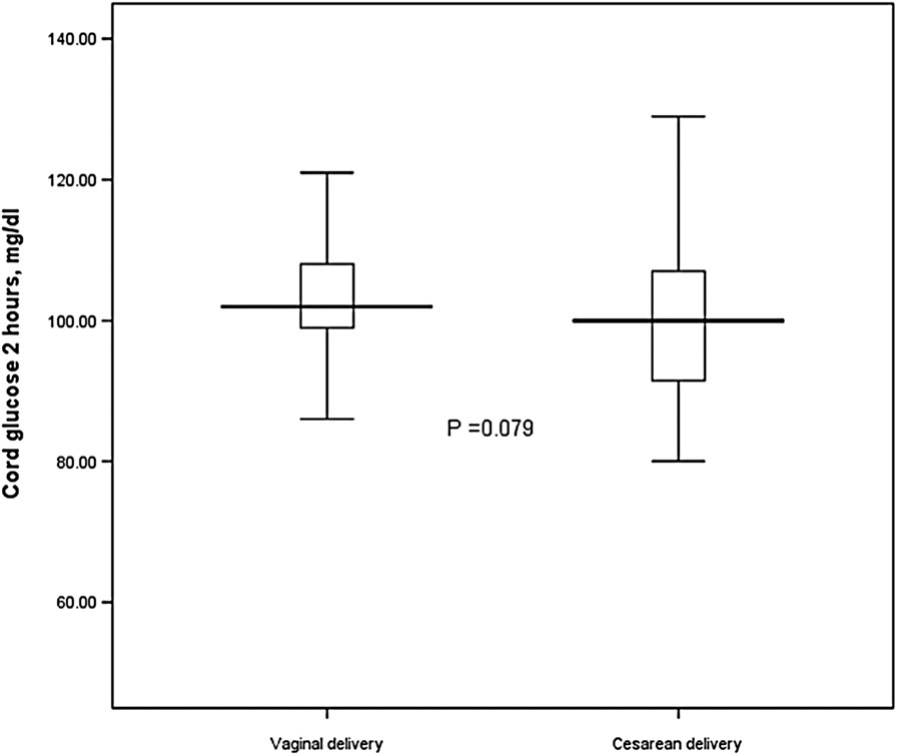
Newborn blood glucose levels at 2 hours after delivery in vaginal and cesarean deliveries.

There were no significant differences in maternal blood glucose levels at delivery and at 2 hours after delivery in the two groups of women. However, cord blood glucose levels were significantly lower in women who delivered by cesarean than in those who delivered vaginally (99.8 ± 20.6 vs. 106.8 ± 11.1 mg/dl, P = 0.026). There was no significant difference in neonatal blood glucose levels at 2 hours after delivery between women who delivered by cesarean and those who delivered vaginally (97.8 ± 16.7 vs. 102.1 ± 9.6, P = 0.110) (Table 2 and Figures 1 and 2).

**Table 2:** Maternal and neonatal blood glucose levels in vaginal and cesarean deliveries in Khartoum, Sudan

**Variables**	**Vaginal delivery**	**Cesarean delivery**	**P**
	**(n = 55)**	**(n = 55)**	
Maternal glucose at delivery, mg/dl	107.9 (14.6)	107.1 (17.0)	0.806
Maternal glucose 2 hours following delivery, mg/dl	101.5 (11.8)	103.4 (15.6)	0.450
Cord glucose, mg/dl	106.8 (11.1)	99.8 (20.6)	0.026
Infant blood glucose at 2 hours, mg/dl	102.1 (9.6)	97.8 (16.7)	0.110

In linear regression, the factors that were significantly associated with mean cord blood glucose levels were cesarean delivery (-6.475 mg/dl, P = 0.013) and maternal glucose at the time of delivery (+0.619 mg, P < 0.001, Table 3). The factors that were significantly associated with mean newborn blood glucose levels at 2 hours after delivery were cord blood glucose (+0.954 mg, P < 0.001) and maternal glucose at 2 hours after delivery (+0.161, P = 0.004, Table 4).

**Table 3:** Factors affecting cord blood glucose levels using multiple linear regressions

**Variable**	**Coefficient**	**Standard error**	**P-value**
Maternal age	0.429	0.357	0.232
Parity	─ 1.017	1.128	0.369
Gestational age	─ 0.447	1.438	0.757
Body mass index	─ 0.904	─ 0.103	0.176
Cesarean delivery	─ 6.475	─ 0.194	0.013
Maternal glucose at delivery	0.619	0.590	< 0.001
Birth weight	6.370	6.411	0.323
Fetal gender	4.475	0.140	0.065

**Table 4:** Factors affecting newborn blood glucose levels at 2 hours of age using multiple linear regressions

**Variable**	**Coefficient**	**Standard error**	**P-value**
Maternal age	0.014	0.114	0.683
Parity	0.004	0.358	0.917
Gestational age	0.001	0.448	0.983
Body mass index	─ 0.017	0.206	0.541
Cesarean delivery	0.001	1.801	0.992
Maternal glucose at delivery	─ 0.105	0.055	0.101
Birth weight	0.043	2.020	0.165
Fetal gender	─ 0.050	0.791	0.081
Cord glucose	0.954	0.031	< 0.001
Maternal glucose at 2 hours	0.161	0.053	0.004
Time to start feeding	─ 0.016	0.074	0.591
Duration of maternal fasting	0.029	0.314	0.651

A similar finding was reported by Melkie and his colleagues who found that cord blood glucose levels were significantly higher in vaginally-delivered newborns than in cesarean-delivered newborns at delivery, with no significant difference at 2 hours after delivery [7]. Recently, cord blood glucose levels were reported to be lower in cesarean delivery than in vaginal delivery [6]. However, the change in blood glucose levels over the first 2 hours of life was significantly higher in cesarean delivery vs. vaginal delivery (glucose levels alone were not different between the two groups). Additionally, cord blood glucose levels significantly affected the change in blood glucose levels over the first 2 h after delivery [6]. Similarly, a previous study showed that cord blood glucose values were significantly higher than those of newborns delivered by cesarean. These findings suggest that stress results in release of catecholamines during vaginal delivery [11,12].

The duration of fasting had no significant association with mean maternal blood glucose levels at delivery and at 2 hours following delivery. Maternal blood glucose levels at delivery were significantly associated with maternal blood glucose levels at 2 hours after delivery (+852 mg, P < 0.001).

In vaginal delivery, there was no correlation between cord blood glucose levels and newborn blood glucose levels 2 hours after delivery (*r* = 0.257, P = 0.056) and the duration of labor (*r* = 0.238, P = 0.078).

During fetal life, cord blood glucose concentrations are correlated with maternal blood glucose concentrations [13]. Fasting time and the catecholamines released during vaginal delivery are possible mechanisms for this difference in blood glucose levels [6]. Interestingly, in the current study, although maternal blood glucose levels were not different between women who delivered vaginally and those who delivered by cesarean, both cord blood glucose and newborn blood glucose levels 2 hours after delivery were associated with maternal blood glucose levels. Furthermore, in spite of a longer fasting time in women who delivered by cesarean than in those who delivered vaginally, fasting time was not significantly associated with maternal blood glucose levels. Therefore, newborn blood glucose was correlated with maternal blood glucose, which was not correlated with fasting time, which was assumed to be the more plausible explanation before [6].

In the current study, there was no correlation between blood glucose levels and the duration of labor in women who delivered vaginally. The stress of labor on the mother and newborn increases maternal and fetal catecholamines [14,15]. Catecholamine release is higher during normal vaginal delivery than during cesarean delivery [14]. This process forms an essential part of adaptation of the fetus to the extra-uterine environment [16].

## Conclusions

In this study cord blood glucose levels are significantly lower in cesarean-delivered newborns than in vaginally-delivered newborns. In addition, cord blood glucose levels are significantly associated with cesarean delivery and maternal blood glucose levels at delivery.

## Competing interest

The authors declare that they have no competing interests.

## Authors’ contributions

SMH and IA designed the study. YS, JAB and DAR conducted the clinical work. IA, JAB and IA performed the statistical analyses. All of the authors read and approved the final manuscript.
